# MethGo: a comprehensive tool for analyzing whole-genome bisulfite sequencing data

**DOI:** 10.1186/1471-2164-16-S12-S11

**Published:** 2015-12-09

**Authors:** Wen-Wei Liao, Ming-Ren Yen, Evaline Ju, Fei-Man Hsu, Larry Lam, Pao-Yang Chen

**Affiliations:** 1 Institute of Plant and Microbial Biology, Academia Sinica, Taipei 11529, Taiwan; 2 Department of Electrical and Computer Engineering, Carnegie Mellon University, Pittsburgh, PA 15213, USA; 3 Department of Molecular, Cell and Developmental Biology, University of California, Los Angeles, CA 90095, USA

## Abstract

**Background:**

DNA methylation is a major epigenetic modification regulating several biological processes. A standard approach to measure DNA methylation is bisulfite sequencing (BS-Seq). BS-Seq couples bisulfite conversion of DNA with next-generation sequencing to profile genome-wide DNA methylation at single base resolution. The analysis of BS-Seq data involves the use of customized aligners for mapping bisulfite converted reads and the bioinformatic pipelines for downstream data analysis.

**Results:**

Here we developed MethGo, a software tool designed for the analysis of data from whole-genome bisulfite sequencing (WGBS) and reduced representation bisulfite sequencing (RRBS). MethGo provides both genomic and epigenomic analyses including: 1) coverage distribution of each cytosine; 2) global cytosine methylation level; 3) cytosine methylation level distribution; 4) cytosine methylation level of genomic elements; 5) chromosome-wide cytosine methylation level distribution; 6) Gene-centric cytosine methylation level; 7) cytosine methylation levels at transcription factor binding sites (TFBSs); 8) single nucleotide polymorphism (SNP) calling, and 9) copy number variation (CNV) calling.

**Conclusions:**

MethGo is a simple and effective tool for the analysis of BS-Seq data including both WGBS and RRBS. It contains 9 analyses in 5 major modules to profile (epi)genome. It profiles genome-wide DNA methylation in global and in gene level scale. It can also analyze the methylation pattern around the transcription factor binding sites, and assess genetic variations such as SNPs and CNVs. MethGo is coded in Python and is publically available at http://paoyangchen-laboratory.github.io/methgo/.

## Background

Cytosine methylation is a crucial epigenetic modification involved in numerous biological processes, including transcriptional regulation, cell differentiation, and X-chromosome inactivation [1]. It is very important in the development in plants, animals, and human [2,3]. Many human diseases and cancers have been found to be associated with abnormal DNA methylation [4].

To evaluate DNA methylation, bisulfite treatment of genomic DNA has been widely used to convert unmethylated cytosines (Cs) to uracils while methylated Cs remain unconverted [5]. After PCR amplification, the converted Cs (to uracils) will be replaced by thymines (Ts). By comparing the bisulfite-converted sequences with the unconverted, the methylation status can be revealed.

Sodium bisulfite treatment coupling with high throughput sequencing (BS-seq) makes it possible to profile genome-wide DNA methylation in single base resolution [5,6]. The two major sequencing strategies for BS-seq are, reduced representation bisulfite sequencing (RRBS) which uses restriction enzymes to digest genomic DNA and size selects CpG-rich areas of genome [7], and whole-genome bisulfite sequencing (WGBS) which investigates all cytosines in the genome, and is state-of-the-art profiling method for genome-wide DNA methylation [8,9]. Both methods are used to profile the epigenomes of cell lines and tissues by large consortiums such as the ENCODE project [10], NIH Roadmap Epigenomics project [11], and The Cancer Genome Atlas (TCGA) [12].

The first step to process BS-seq data is to align the BS reads to the reference genome. Aligners such as Bowtie2[13] and SOAP [14] are not applicable since the C-to-T conversion in the BS reads are incorrectly treated as mismatches for mapping penalty. Customized bisulfite aligners such as BS-Seeker2 [15] were introduced to efficiently perform genome indexing, read mapping and methylation level calling.

After alignment, further bioinformatics steps are required for extracting biologically meaningful information. Several tools for such post-alignment analysis including Kismeth [16], Bis-SNP [17], GBSA [18], Repitools [19], and ReadDepth [20]. As these tools are designed for specific analyses, there is a lack of platforms providing a comprehensive overview of the BS-Seq data covering both genomic and epigenomic analyses.

In this paper we present MethGo, a post-alignment tool consisting of 9 analyses in 5 functional modules for processing and analyzing BS-Seq alignments. MethGo provides coverage distribution across all methylation sites, global methylation states and methylation levels according to several defined regions, such as promoter, gene body or transcription factor binding sites (TFBSs). In addition to DNA methylation, MethGo also provides the information of genetic variations including CNV calling and SNP calling. MethGo produces high quality figures and tables for data presentation that are ready for scientific publication.

## Implementation

**Figure 1:**
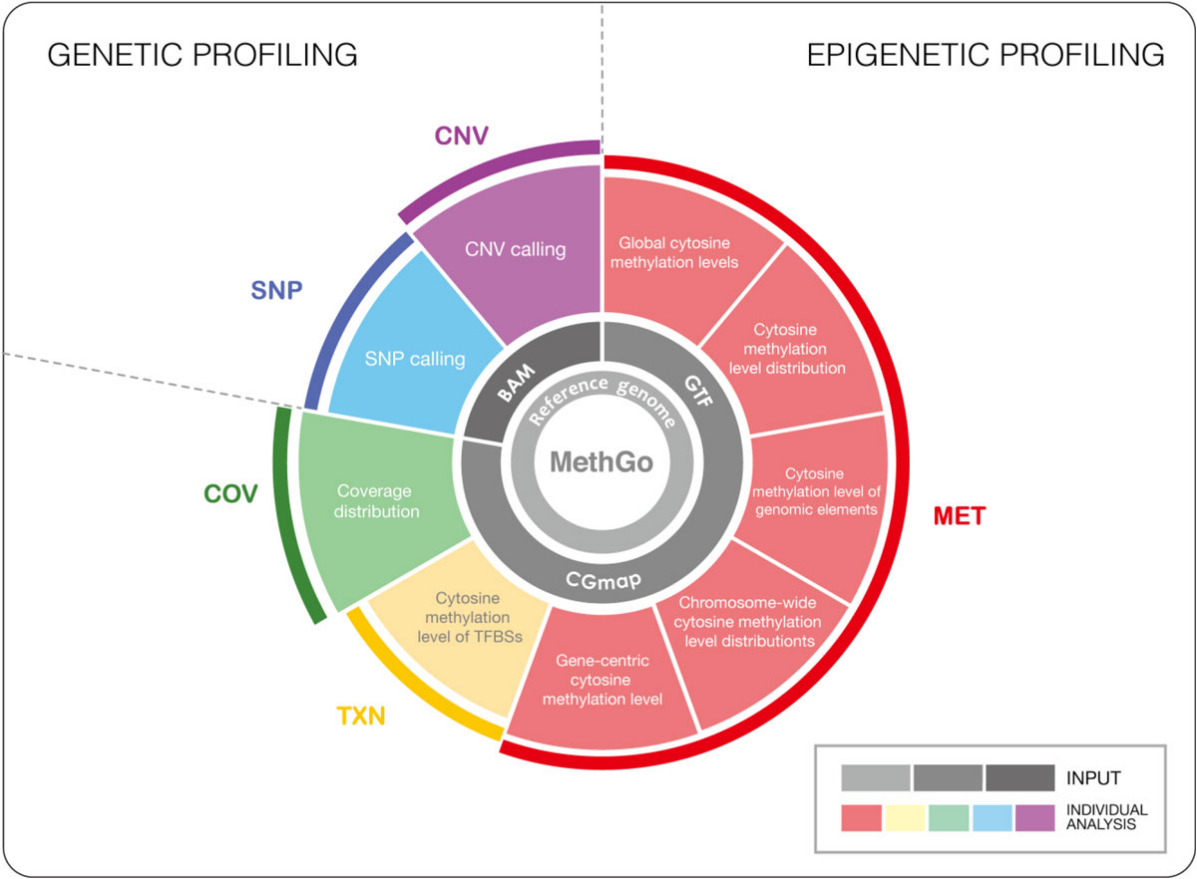
**Overview of MethGo software pipeline**. MethGo is a comprehensive tool for analyzing post-alignment bisulfite reads. MethGo consists of five modules for investigating DNA methylation as well as genetic variations.

MethGo is a Python software that takes the alignment file from both WGBS and RRBS as the input data. It consists of three modules for methylation analysis and two modules to detect genetic variations (Figure 1, and see Additional file 1 and Additional file 2 for the description of the modules): COV module generates coverage distribution for methylation sites. MET module provides global cytosine methylation levels, cytosine methylation level distributions, cytosine methylation levels of genomic elements, chromosome-wide cytosine methylation level distributions, and gene-centric cytosine methylation levels. TXN module plots the methylation level relative to TFBSs. The SNP module detects SNPs and the CNV module detects CNVs across the genome.

Here we describe the 5 modules in more details:

### COV: coverage distribution of methylation sites

**Figure 2:**
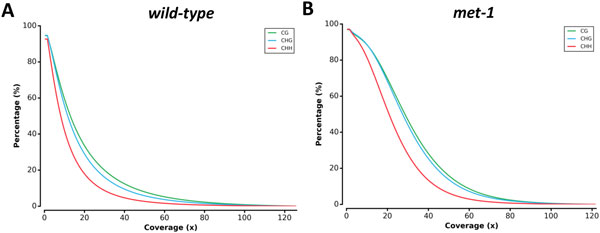
**Coverage distribution of *Arabidopsis *WGBS**. A. wild-type. B. *met-1*. Reverse cumulative plot with x-axis representing the coverage and y-axis representing the percentage of sites across genome. CG/CHG/CHH contexts are indicated by green/blue/red.

Coverage of the methylation sites is a factor for evaluating the quality of sequencing data. Sites with high coverage are likely to provide accurate methylation status estimation. The COV module extracts the coverage for each cytosine from post-alignment data (i.e., CGmap) and generates reverse cumulative plot for methylation sites by the genomic contexts (CG, CHG and CHH, H refers to A, C, or T). For example, Figure 2 shows the coverage distribution of two *Arabidopsis *methylomes. In the WT methylome approximately 20% of the genome are covered with 20× depth of sequencing, whereas the *met1 *methylome shows ~70% of the genome are covered. The coverage plot helps user to evaluate the quality of sequencing data and defines the cutoff for reads depth.

### MET: methylation profiling

**Figure 3:**
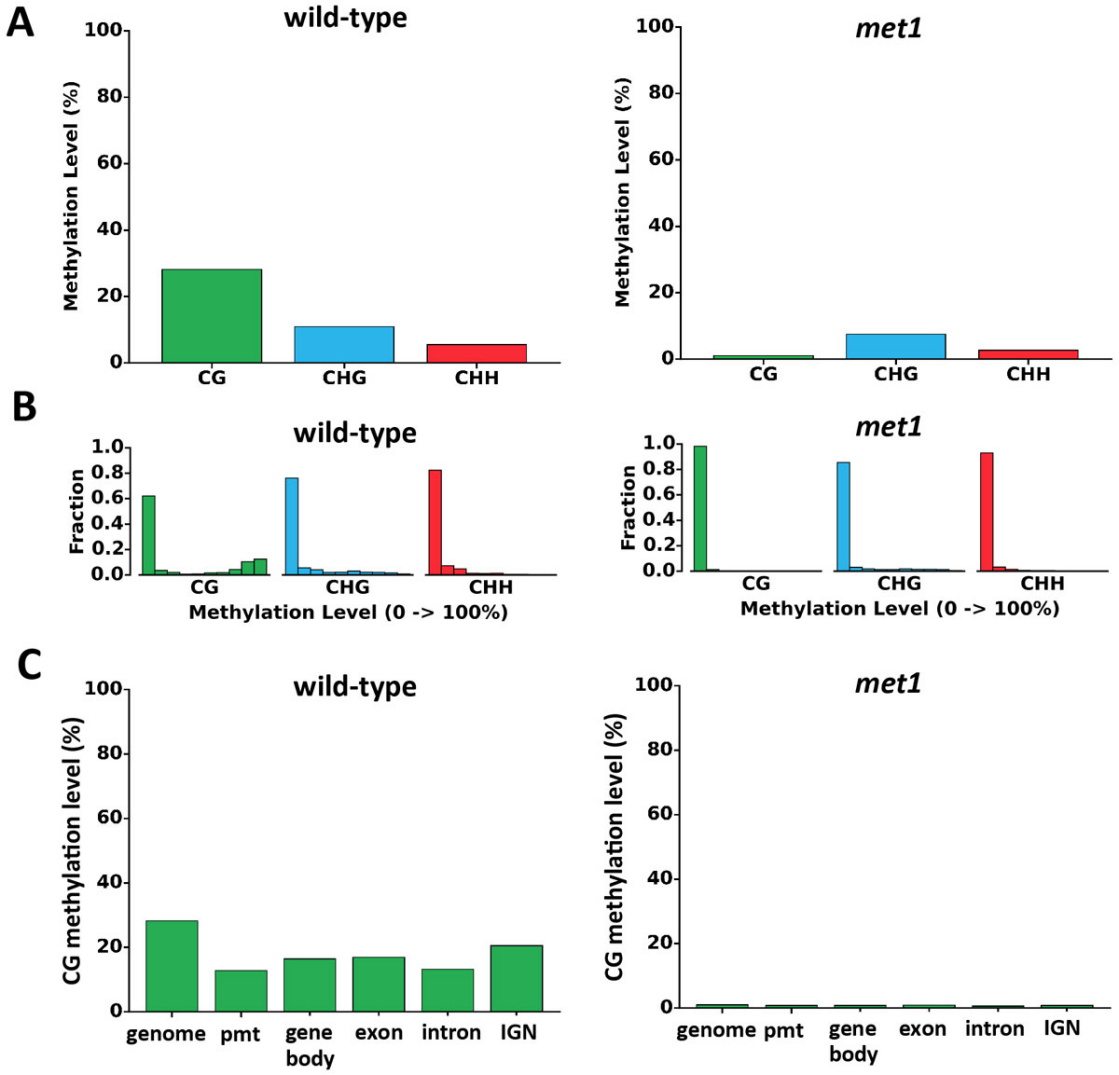
**DNA methylation profiles in wild-type and *met1 *mutant *Arabidopsis***. A. Global cytosine methylation levels. B. Distribution of cytosine methylation. The x-axis represents methylation levels binned in ten increment of 10% (i.e. 0%-10%, 10%-20%, *etc*.); y-axis is the fraction of total CG/CHG/CHH. C. Plot of CG methylation levels in different genomic elements.

**Figure 4:**
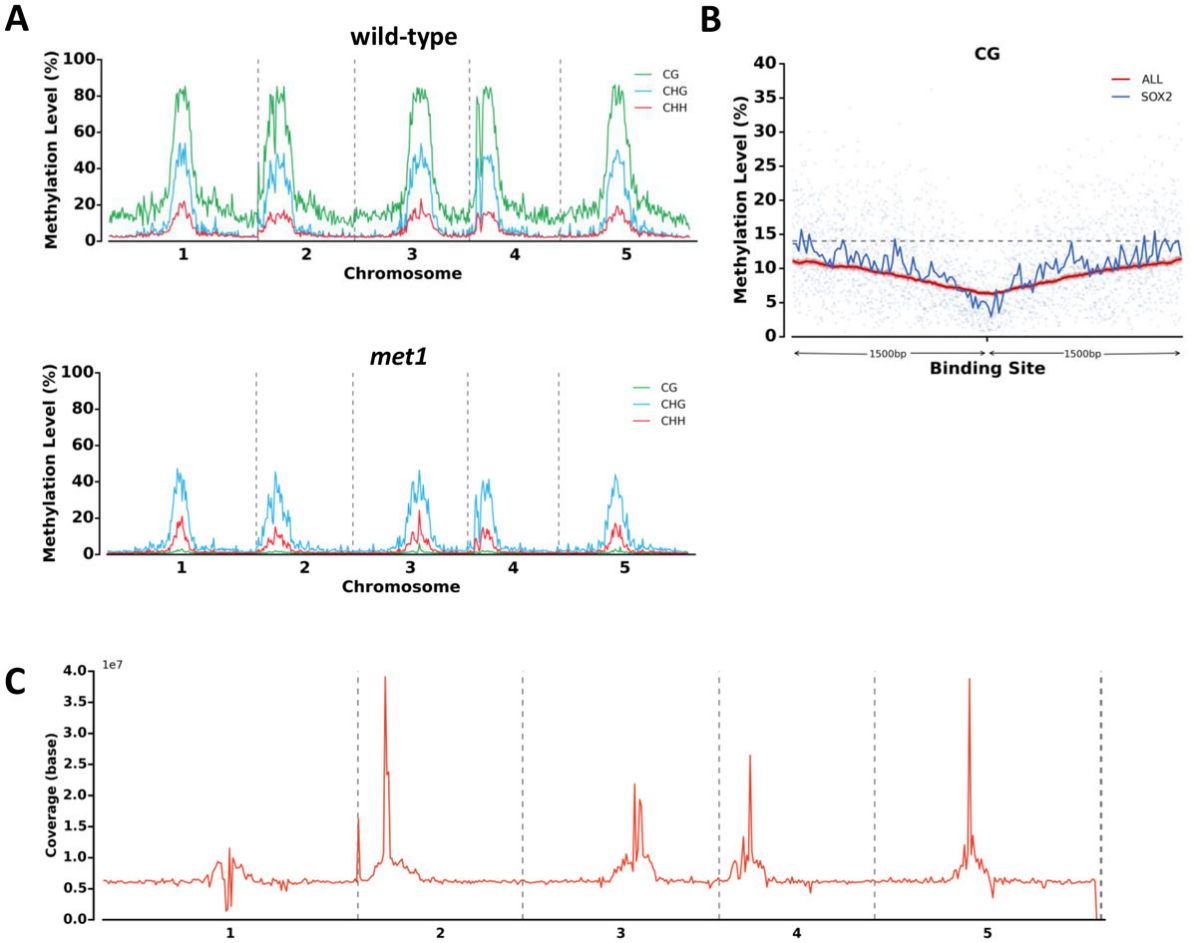
**MethGo profiling of *Arabidopsis *and mouse WGBS**. **A**. Chromosome-wide distribution of cytosine methylation levels in wild-type (top) and *met1 *mutant (bottom) *Arabidopsis*. **B**. Methylation level around TFBS in mouse WGBS. The length of each TFBS is variant, and MethGo takes the center of the sequence for analysis. **C**. CNV on all chromosomes in *Arabidopsis *WGBS.

The MET module takes methylation calls generated from the bisulfite aligner such as BS Seeker 2, and gene annotation file for analyses. Five analyses are carried out in MET module. First, global cytosine methylation level of CG/CHG/CHH are calculated and plotted (Figure 3A). Second, methylation level of sites in each context is calculated and plotted into cytosine methylation level distribution plot (Figure 3B). Third, a genome is further divided into promoter, gene body, exon, intron and intergenic non-coding region (IGN), referring to genomic elements, and generated cytosine methylation level of genomic elements plot (Figure 3C). The promoter is defined as the region 1,000 bp upstream of transcription start site (TSS), and gene body is defined as the region between TSS and transcription termination sites (TTS). Fourth, chromosome-wide cytosine methylation level distribution are generated so that user could visualize the methylation level dynamics across each chromosome (Figure 4A). Fifth, MET module profiles gene-centric cytosine methylation level (Additional file 3), a gene whose methylation levels of promoter, gene body, intron and exon are listed in a summary table.

### TXN: evaluating DNA methylation level at transcription factor binding sites

DNA methylation at the TFBSs can interfere with the binding of proteins and hence affects the activation of transcription [21]. The TXN module aims to reveal such a relationship by plotting the DNA methylation level at the TFBS of specific transcriptional factors. The TXN module processes methylation calls and transcription factor binding positions. The methylation levels within 1,500 bp of the TFBSs are averaged over tiling windows (30 bp) and reported in a scatter plot to reveal the methylation pattern around the TFBS (Figure 4B). By comparing the methylation patterns between transcription factors, alternated methylation level around the TFBS suggests the binding of the transcription factor may be associated with DNA methylation.

### CNV: CNV calling

Since BS-seq is DNA-based sequencing, the coverage of the reads (i.e. depth) can be used as a proxy for assessing CNVs. The CNV module extracts the read coverage from the alignments and plots the coverage across the genome (Figure 3C). Genomic regions with large-scale rearrangement such as duplication and deletion are likely to show in coverage plot, amplification and depletion respectively. Therefore, the CNV module is able to detect genome abnormality such as aneuploidy. Regions of continuous depletion or amplification, indicative of genome duplication or deletion, are reported in a text file and represented in a plot of genome-wide copy number.

### SNP: SNP calling

The SNP module identifies both homozygous and heterozygous SNPs from the alignment. The homozygous SNPs are polymorphisms where the majority reads show one dominant allele, which is different from the allele on the reference genome. The heterozygous SNPs are the ones where reads show two major alleles, potentially reflecting the two parental alleles (Additional file 4). In BS-seq, the alignment on the genomic C is not applicable due to the C-to-T conversion, so instead the alignment on the other strand G is used for SNP calling.

## Results and discusssion

### Feature evaluation with other major analyzers

We examined the functional features of MethGo together with five major post-alignment tools for BS-seq analysis, namely Bis-SNP, Kismeth, GBSA, Repitools, and ReadDepth. (see Table 1 for a summary of their functional features). Bis-SNP is written in Java, provides methylation levels for each cytosine and calls SNPs from BS-seq data. Kismeth is a web-based tool, which calculates global methylation levels and provides platform for nucleotide-resolution methylation status visualization. GBSA is a tool written in Python and provides sequencing quality assessment, gene-centric methylation level, functional data management and visualization of methylation in nucleotide resolution. Repitools is an R package for the analysis of enrichment-based assay and displays the distribution of enriched DNA across the genome followed by visualizing and summarizing the interaction between epigenetic mark and gene expression. ReadDepth is also an R package to detect CNVs by measuring the depth of coverage in the sequencing data. MethGo provides 9 analyses for both epigenetic and genetic profiling, including coverage distribution, global cytosine methylation level, cytosine methylation level distribution, chromosome-wide cytosine methylation level distribution, cytosine methylation level of genomic elements, gene-centric cytosine methylation level, cytosine methylation level of TFBSs, CNV calling, and SNP calling. Altogether, MethGo includes the functions such as cytosine methylation level distribution, cytosine methylation level of genomic elements, chromosome-wide cytosine methylation level distribution and cytosine methylation level of TFBSs, which are not included in Bis-SNP, GBSA, Kismeth, Repitools, and ReadDepth. In addition, MethGo is the only tool to profile both SNPs and CNVs.

**Table 1:** Summary of bioinformatic tools for data analysis using aligned BS-seq.

	MethGo	**Kismeth**[16]	**Bis-SNP**[17]	**GBSA**[18]	**Repitools**[19]	**ReadDepth**[20]
Programming Language	Python	unknown	Java	Python	R	R
Operating System	Windows/ Unix	web	Windows/ Unix	Windows/ Unix	Windows/ Unix	Unix
Interface	Command-line	GUI	Command-line	GUI/ Command-line	Command-line	Command-line
Coverage distribution	Yes (*F)	-	-	-	Yes	-
Global cytosine methylation level	Yes (*F)	Yes	-	-	-	-
Cytosine methylation level distribution	Yes (*F)	-	-	-	-	-
Cytosine methylation level of genomic elements	Yes (*F)	-	-	-	-	-
Chromosome-wide cytosine methylation level distribution	Yes (*F)	-	-	-	-	-
Gene-centric cytosine methylation level	Yes (*T)	-	-	Yes	-	-
Cytosine methylation level of TFBSs	Yes (*F)	-	-	-	-	-
SNP calling	Yes (*T)	-	Yes	-	-	-
CNV calling	Yes (*F)	-	-	-	-	Yes

Main functions of tool	Methylation profiling and extracting genetic variation information from bisulfite sequencing data	Global methylation levels calculation and visualization at nucleotide resolution	SNP calling	Gene-centric methylation level scoring and visualizetion	Enrichment based epigenomic data analysis such as coverage distribution of CpG sites	CNV calling

*F: the output file is figure; *T: the output file is table.

### Demonstrating COV, MET, CNV and SNP modules with *Arabidopsis *WGBS data

In order to demonstrate MethGo on real data, we downloaded and processed WGBS data of wild-type and *met1 *mutant of *Arabidopsis *[22]. MET1 is methyltransferase 1, which controls faithful maintenance of cytosine methylation primarily at CG sites. After mapping with BS aligner, BS-Seeker2, the output was loaded into MethGo for processing. COV module outputs reverse cumulative plot of coverage distribution. Different sequencing samples show different coverage distribution due to sequencing depth of data. (Figure 2A, B).

As for DNA methylation profiling with MET module, the CG methylation in *met1 *mutant is much lower compared to WT (Figure 3A). The *met1 *mutant almost abolishes the CG methylation with relatively less effect on CHG and CHH contexts. The cytosine methylation distribution plots show the methylation distribution of cytosine sites in all three contexts. As shown in Figure 3B, the CG methylation shows a bimodal distribution where most sites are either in low or high methylation. The CHG and CHH sites are generally weakly methylated. The cytosine methylation level of genomic elements plots showed the average methylation level in genome, promoter, gene body, exon, intron, and intergenic regions by CG, CHG, and CHH contexts (Figure 3C and Additional file 5). Compared with other regions, the methylation level in promoters is lower due to facilitation of protein binding. The chromosome-wide cytosine methylation level distribution showed the landscape of DNA methylation throughout a genome (Figure 4A). The plots showed that in *Arabidopsis*, the methylation levels are higher near the pericentromeric regions in all contexts. The MET module also generates gene-centric cytosine methylation levels for each gene for wild-type *Arabidopsis *(Additional file 3). The CNV module profiles genome-wide CNVs (Figure 4C). Peri-centromeric regions of all 5 chromosomes show high coverage due to the presence of repetitive sequence. The SNP calling module generates tabular file of homozygous and heterozygous SNPs, which helps researchers to investigate potential mutations or serves as a marker for genotyping (Additional file 6 and Additional file 7).

### TXN module demonstration with mouse WGBS data

We downloaded WGBS data of mouse primordial germ cells to demonstrate TXN module of MethGo [23]. The accessibility of TFBS is important for gene regulation and TFBS should be exploited of DNA methylation. TXN module plots methylation levels of specific transcription factor of interest and an average methylation level for comparison. In Figure 4B, SOX2 is a transcription factor regulating cell pluripotency [24], and its corresponding TFBS shows significant decrease of methylation relative to average of all TFBSs.

## Conclusions

We presented MethGo, specifically for analyzing post-alignment from BS-seq. In comparison with other popular similar tools, MethGo is a streamlined tool capable of profiling both genome-wide DNA methylation and genetic variations. It also generates high resolution plots. MethGo comes with a user-friendly manual and tutorials with examples for biologists to evaluate DNA methylation. The MethGo installation guide and module requirements can be found in Additional file 8. We have made this tool publicly available for the community.

## Competing interests

The authors declare that they have no competing interests.

## Authors' contributions

PC conceived the project. WL, EJ and LL implemented the software. MY wrote the manuscript. FH, EJ, LL and PC edited the manuscript. All authors have read and approved the final manuscript.

## Supplementary Material

Additional file 1 **Detailed description of implementation**. This file contains information on the implementation for all the modules. Click here for file

Additional file 2 **File format of input and output**. Table of file format of input and output required for MethGo modules. Click here for file

Additional file 3 **DNA methylation of genes in wild-type *Arabidopsis***. Average DNA methylation levels of regions related to genes, including promoter, gene body, exon, and intron. Click here for file

Additional file 4 **Heterozygous and homozygous SNP**. A. The illustration of heterozygous and homozygous SNP. B. Screenshot of heterozygous SNP. There are two different alleles comparing to reference genome (bottom). C. Screenshot of homozygous SNP. There is one allele different from the reference genome (bottom). Click here for file

Additional file 5 **Non-CG methylation levels in different genomic elements in *Arabidopsis***. A. wild-type. B. *met1*. Click here for file

Additional file 6 **Homozygous SNPs**. This file contains homozygous SNP calling of WT *Arabidopsis*. Click here for file

Additional file 7 **Heterozygous SNPs**. This file contains heterozygous SNP calling of WT *Arabidopsis*. Click here for file

Additional file 8 **Software installation guide and requirements**. This file contains the MethGo installation guide and module requirements. Click here for file

## Declarations

Publication charges for this article have been funded by a grant from Academia Sinica to P.-Y. C.

This article has been published as part of *BMC Genomics *Volume 16 Supplement 12, 2015: Joint 26th Genome Informatics Workshop and 14th International Conference on Bioinformatics: Genomics. The full contents of the supplement are available online at http://www.biomedcentral.com/bmcgenomics/supplements/16/S12.
