# Supplementary material for: MethGo: a comprehensive tool for analyzing whole-genome bisulfite sequencing data
Source: BMC Genomics. 2015 Dec 9;16(Suppl 12):S11. doi: 10.1186/1471-2164-16-S12-S11 (PMC4682368; doi:10.1186/1471-2164-16-S12-S11)
Supplement: Additional file 1 — Detailed description of implementation. This file contains information on the implementation for all the modules. [file 1471-2164-16-S12-S11-S1.pdf]

Detailed description of implementation

COV module

The COV module uses information from methylation calls in the form of a CGmap to calculate coverage of all methylation sites in three contexts. The inputs are a CGmap, and reference genome. For each cytosine position, the coverage is determined, the first to identify all the cytosine sites from reference genome, and the second to extract coverage information from the CGmap file. The reverse cumulative plot for coverage distributions is reported.

MET module

The MET module uses information from methylation calls in the form of a CGmap and gene annotations to identify gene methylation levels and their respective promoter methylation levels. The inputs are a CGmap, gene annotation file, and reference genome. For each chromosome in the reference genome, two dictionaries are generated, the first to mark the positions of methylation for each methylation contexts, and the second to note the methylation levels at each point of methylation. A list of genes and their bounds are generated from the gene annotation file in General Transfer Format (GTF). For each gene, all the methylation levels of 3 contexts within the gene bounds are averaged to produce its gene body methylation level. Promoters with a size specified by the user (default is 1000 bp) are then analyzed in the same manner to produce promoter methylation levels for each gene. Based on methylation contexts, results of gene methylation levels sorted by their gene ID are outputted in a text file.

TXN module

The TXN module plots the methylation levels adjacent to the transcription factor binding sites (TFBSs) from methylation calls in the form of a CGmap and TFBS annotations. The inputs are a CGmap and a list of transcription factors. For each TFBS, the methylation levels of sites within 1,500 bp are averaged over tiling windows (30 bp). The methylation levels distributions are reported in a scatter plot with smooth curve.

CNV module

The CNV module investigates the number of copies of genes in the genotype of an individual to find areas in the genome likely to have large-scale genome rearrangement. Inputs include a sorted BAM file and reference genome index. The PySAM pileups method is used in order to obtain the number of bases for reads at each position in the reference genome. Using the window size given by the user (default is 200,000), all the bases of the reads at the positions within each window are summed up. The standard Z score is calculated and converted to a P-value for each window. If there are 3 windows in a row (user can change this default setting) and all their P-values are smaller than a given threshold (default is 0.05), then this region is considered a CNV.

SNP module

The PySAM pileups method is used in order to obtain the alleles for reads at each position in the reference genome. Allele counts are then determined for each position, and if the coverage, or number of reads present at that position, exceeds a given amount (default is 5), the alleles at that position are analyzed for the presence of a SNP. Homozygous SNPs are considered to have occurred at positions in which an allele exists with a frequency higher than the given major allele frequency (default is 0.9) in the reads that overlap at that position. Additionally, the major allele needs to differ from the allele in the reference genome at that position. Heterozygous SNPs are considered to have occurred when two alleles occur with frequencies in the reads within a range close to 0.5. A buffer is set (default is 0.1) around 0.5 for the frequencies of the two alleles to be within (so default frequencies are 0.4-0.6) to be considered a heterozygous SNP.
