# Supplementary material for: MethGo: a comprehensive tool for analyzing whole-genome bisulfite sequencing data
Source: BMC Genomics. 2015 Dec 9;16(Suppl 12):S11. doi: 10.1186/1471-2164-16-S12-S11 (PMC4682368; doi:10.1186/1471-2164-16-S12-S11)
Supplement: Additional file 2 — File format of input and output. Table of file format of input and output required for MethGo modules. [file 1471-2164-16-S12-S11-S2.pdf]

Additional File 2. File format of input and output

Module	SNP	CNV	MET	COV	TXN
**Input**	-Sorted BAM<br>-Reference genome	-Sorted BAM<br>-Reference genome index	-Gene annotation file<br>-Reference genome<br>-CG map	-Sorted BAM<br>-Reference genome	-CGmap<br>-TFBSs
**Output**	-SNP table	-CNV table<br>-CNV plot	-Global cytosine methylation level plot<br>-Cytosine methylation level distribution plots<br>-Cytosine methylation level plot of different genomic elements<br>-Chromosome-wide cytosine methylation level distribution plot<br>-Gene-centric methylation level tabl	-Reverse cumulative plot	-Cytosine methylation levels plot around TFBS
**Parameters**	-Coverage (minimum number of reads)<br>-Allele frequency to be considered homozygous SNP<br>-Buffer around 0.5 to be considered heterozygous SNP	-Window size (number of bases for reads to be summed over)<br>-Probability (probability cutoff for window to be considered a CNV)<br>-Window number (number of windows for moving average to be calculated over)	-Depth (minimum number of reads)<br>-Promoter size (number of bases)<br>-Methylation context		
