# Supplementary material for: MethGo: a comprehensive tool for analyzing whole-genome bisulfite sequencing data
Source: BMC Genomics. 2015 Dec 9;16(Suppl 12):S11. doi: 10.1186/1471-2164-16-S12-S11 (PMC4682368; doi:10.1186/1471-2164-16-S12-S11)
Supplement: Additional file 4 — Heterozygous and homozygous SNP. A. The illustration of heterozygous and homozygous SNP. B. Screenshot of heterozygous SNP. There are two different alleles comparing to reference genome (bottom). C. Screenshot of homozygous SNP. There is one allele different from the reference genome (bottom). [file 1471-2164-16-S12-S11-S4.pdf]

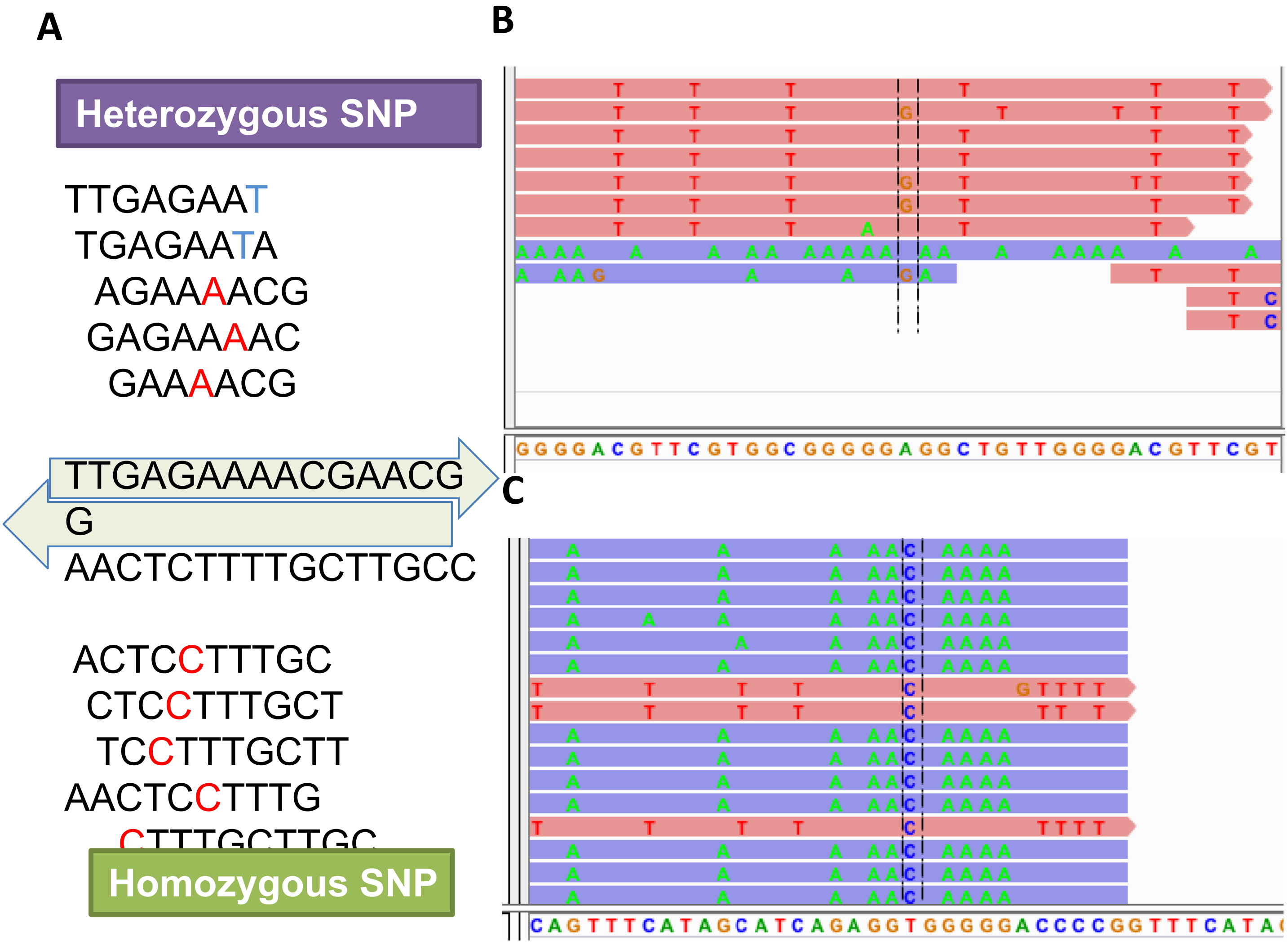


**Additional file 4: Heterozygous and homozygous SNP.**

A. The illustration of heterozygous and homozygous SNP. B. Screenshot of heterozygous SNP. There are two different alleles comparing to reference genome (bottom). C. Screenshot of homozygous SNP. There is one allele different from the reference genome (bottom).
