# Supplementary material for: MethGo: a comprehensive tool for analyzing whole-genome bisulfite sequencing data
Source: BMC Genomics. 2015 Dec 9;16(Suppl 12):S11. doi: 10.1186/1471-2164-16-S12-S11 (PMC4682368; doi:10.1186/1471-2164-16-S12-S11)
Supplement: Additional file 5 — Non-CG methylation levels in different genomic elements in Arabidopsis. A. wild-type. B. met1. [file 1471-2164-16-S12-S11-S5.pdf]

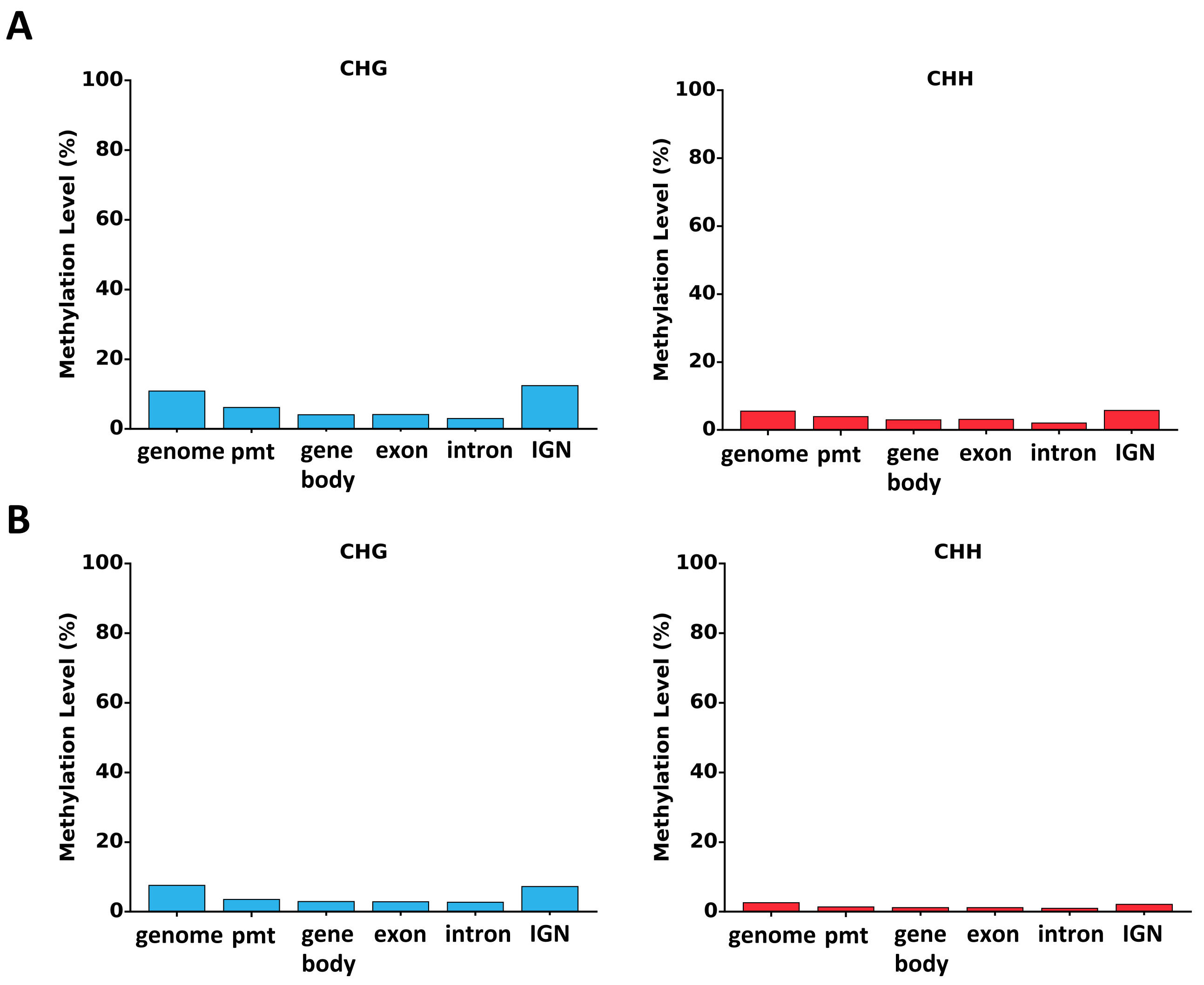

Additional file 5: Non-CG methylation levels in different genomic elements in *Arabidopsis*.

A. wild-type. B. *met1*.
