# Supplementary material for: MethGo: a comprehensive tool for analyzing whole-genome bisulfite sequencing data
Source: BMC Genomics. 2015 Dec 9;16(Suppl 12):S11. doi: 10.1186/1471-2164-16-S12-S11 (PMC4682368; doi:10.1186/1471-2164-16-S12-S11)
Supplement: Additional file 8 — Software installation guide and requirements. This file contains the MethGo installation guide and module requirements. [file 1471-2164-16-S12-S11-S8.pdf]

Installation

The following are step-by-step instructions to guide you go through the installation process. For an up-to-date installation guide, please visit the documentation at http://methgo.readthedocs.org/.

1. MethGo depends on SAMtools and BEDtools, so please make sure you already have them on your server.

2. Obtain Python 2.7 and virtualenv.

3. Create a virtual environment somewhere on your disk, and then activate it.

```
$ virtualenv methgo_env
$ cd methgo_env
$ source bin/activate
```

4. Download the source code and install the requirements.

```
$ git clone https://github.com/paoyangchen-laboratory/methgo.git
$ cd methgo
$ chmod +x methgo
$ echo 'export PATH=$PATH:~/.local/methgo' >> ~/.bashrc
$ source ~/.bashrc
$ pip install -r requirements/base.txt
$ pip install -r requirements/addition.txt
```

`pip` will install the following packages:

- NumPy
- SciPy
- matplotlib
- pandas
- PySAM == 0.8.0
- Biopython
- pyfasta
- Cython
- pybedtools
